# Association of social vulnerability with access to treatment in ovarian cancer patients

**DOI:** 10.1016/j.gore.2025.101808

**Published:** 2025-07-20

**Authors:** Nikita Bastin, Marc Robinson, Amir Javid, Lauren S. Prescott, Alaina J. Brown

**Affiliations:** a Vanderbilt University School of Medicine, Nashville, TN, USA; b Vanderbilt University Medical Center, Nashville, TN, USA

**Keywords:** Ovarian cancer, Social vulnerability index, Access to treatment, Disparities, Palliative care

## Abstract

**Objectives:**

To understand disparities in access to treatment for ovarian cancer patients.

**Methods:**

A retrospective review of patients who had surgery for ovarian cancer at a comprehensive cancer center from 2018 to 2024 was completed. Social Vulnerability index quartiles generated by patients’ zip codes were used as measures of social vulnerability. Social vulnerability encompasses four themes, including socioeconomic status, household characteristics, racial/ethnic minority status, and housing type and transportation. The primary outcome was time to treatment initiation. Secondary outcomes included time to chemotherapy, time to surgery, time to palliative care treatment, and progression-free survival. Logistic regression was used to assess the relationship between social vulnerability and treatment access.

**Results:**

Among 166 ovarian cancer patients, there was a trend towards increased time from diagnosis to treatment amongst patients with the highest social vulnerability (median 40 days) when compared to those with lower social vulnerability (median 22 – 27 days). When examining specific treatments received, patients with lower social vulnerability were more likely to experience shorter times to chemotherapy (median 29.5–32 days) when compared to patients with higher social vulnerability (median 41 – 45 days). Patients with the highest social vulnerability experienced the longest time to surgery (median 128 days) amongst all quartiles. There was a trend towards shorter times from diagnosis to palliative care consultation for patients of higher social vulnerability.

**Conclusions:**

Higher social vulnerability was associated with longer times to curative treatment and shorter times to palliative care consultation among ovarian cancer patients. Further study in more diverse patient populations is necessary to ensure the equitable delivery of care in ovarian cancer.

## Highlights

• Ovarian cancer patients with higher social vulnerability may have worse oncologic outcomes, including decreased survival.
• This is the first study to examine access to treatment in ovarian cancer patients according to social vulnerability.
• Patients with high social vulnerability experience longer times to curative treatment and shorter times to palliative care.
• Social vulnerability may serve as a valuable tool for holistically evaluating a patient’s risk of delay.

## 1 Introduction

Ovarian cancer is a leading cause of cancer-related deaths in women (Key Statistics for Ovarian Cancer, 2025, Surveillance and Program, 2018). It is a disease with notoriously high mortality rates following diagnosis, with a 5-year relative survival rate of 51.6 % (Surveillance and Program, 2018). Significant disparities in ovarian cancer care have been noted according to race and socioeconomic status, with black and lower socioeconomic status patients being more likely to receive guideline-discordant treatment and experience higher mortality (LaRaja et al., 2024, Akinyemiju et al., 2018, Washington et al., 2024).

Multiple hypotheses have been suggested to explain the significant racial disparities in ovarian cancer diagnosis and outcomes. Researchers have explored the impacts of differential access to healthcare, structural racism, and microbiologic tumor differences, among others (Karanth et al., 2019, Mullins et al., 2019, Srivastava et al., 2017, Mei et al., 2023). Identifying factors that contribute to differences in access may help mitigate the observed disparities.

In examining the challenges faced by our patients from a health equity lens, it is evident that patients have multiple identities mediating their interactions with the healthcare system (Simkus et al., 2024). Overlapping systems of discrimination lead to compounded disparities. Social vulnerability provides an appropriately comprehensive measure of healthcare access—it is a multidimensional representation of an individual’s economic, environmental, and sociocultural conditions (CDC/ATSDR Social Vulnerability Index, 2022). Few studies have employed aggregate measures such as social vulnerability to examine ovarian cancer care, with existing analyses focused on mortality rather than access (Karanth et al., 2019, Hicks et al., 2025, Borho et al., 2025). This underscores the need for further investigation into access to ovarian cancer treatment.

A key, underutilized aspect of ovarian cancer care is palliative treatment—studies have shown that black patients and patients of lower socioeconomic status have less access to palliative services across various diagnoses (Salyer et al., 2022, Bowers et al., 2022). An assessment of access to both curative and palliative treatment amongst ovarian cancer patients can provide a more complete picture of how patients navigate their care.

In this retrospective study, we sought to understand disparities in access to treatment for ovarian cancer patients. To our knowledge, this is the first study to examine access to treatment in ovarian cancer patients according to social vulnerability. Excavating such disparities using social vulnerability can help us better quantify how structural inequities shape ovarian cancer care. We hypothesized that patients with greater social vulnerability experience more delays to treatment.

## 2 Methods

### 2.1 Study sample

This study was an Institutional Review Board (IRB) approved retrospective review of patients who had surgery for ovarian cancer at a single comprehensive cancer center from January 1, 2018 to September 1, 2024. Data was collected using our institutional electronic medical record and the Gynecologic Enhanced Recovery After Surgery Database. These dates were chosen as this database was established January 1, 2018. This is an existing database of the Obstetrics & Gynecology Department at the Vanderbilt University Medical Center (VUMC) that compiles demographic, oncologic, and surgical data in patients with gynecological malignancies treated with surgery.

These patient records were reviewed to ensure that all study eligibility criteria were met prior to initiating study enrollment. Inclusion criteria included women age > 18 years with an ovarian cancer diagnosis and surgical care received between January 2018 and September 2024, with the majority of medical care received at VUMC. Exclusion criteria were multiple primary cancers and an inability to obtain information regarding diagnosis or treatment.

Study data were collected and managed using Research Electronic Data Capture tools housed within VUMC (Harris et al., 2009, Harris et al., 2019).

In accordance with the journal’s guidelines, we will provide our data for independent analysis by a selected team by the Editorial Team for the purposes of additional data analysis or for the reproducibility of this study in other centers if such is requested. This study was IRB-exempt (IRB #240808) as determined by the VUMC IRB.

### 2.2 Study measures

#### 2.2.1 Social vulnerability

The Center for Disease Control developed the Social Vulnerability Index to determine which communities may need support prior to, during, and following disasters. The Social Vulnerability Index categorizes 16 U.S. Census variables from the 5-year American Community Survey into four themes—socioeconomic status, household characteristics, racial and ethnic minority status, and housing type and transportation. These themes were grouped together to generate a Social Vulnerability Index score that serves as an aggregate measure of social vulnerability. Social Vulnerability Index scores were generated based on zip codes and automatically sorted into four national quartiles: low, low-medium, medium–high, and high. Lower scores represent less social vulnerability, and higher scores signify more social vulnerability. The 2022 Social Vulnerability Index dataset was used in our analysis, as it represented the midpoint of our study timeline.

#### 2.2.2 Clinical and treatment characteristics

Data was collected from the electronic medical record by study personnel trained in data extraction. Clinical data recorded included: age, race, ethnicity, attending physician, zip code, and insurance status. Oncologic data collected included: histology of cancer, stage of cancer genetic testing and type of surgical resection.

### 2.3 Data collection and analysis

#### 2.3.1 Outcomes

The primary outcome was time to treatment initiation. Time to treatment initiation was defined as the time (in days) between the date of initial presentation of ovarian cancer symptoms and date of treatment initiation.

Secondary outcomes included time to chemotherapy, time to surgery, and time to palliative care consultation. These outcomes were similarly defined as the time (in days) between the date of initial symptomatic presentation and date of corresponding treatment initiation. Secondary outcomes also included Progression-Free Survival. Progression-Free Survival was defined as the time (in days) between the date of treatment initiation and the date of recurrence.

#### 2.3.2 Statistical analysis

All analyses were performed using R 4.3.3 in March 2025 and a p-value less than 0.05 was considered significant.

Descriptive statistics were calculated. Survival analysis was completed using Cox Proportional Hazards Model. Associations between Social Vulnerability Index quartile and time to treatment, including surgery, chemotherapy, inpatient palliative care, and outpatient palliative care, were analyzed using logistic regression.

## 3 Results

### 3.1 Demographics

The patient cohort included 166 patients diagnosed with ovarian cancer and treated with surgery during the study period. Seventy-four (44.6 %) patients were classified as lower social vulnerability and 92 (55.4 %) patients as higher social vulnerability, with patients further subdivided into quartiles. Most patients were white (92.1 %) and insured privately (54.8 %) or through Medicare (40.8 %) (Table 1). Most had advanced stage disease with N = 92 (56.4 %) with stage III and N = 40 (24.5 %) stage IV disease. Most patients underwent genetic testing (83.1 %), and the majority were treated with chemotherapy (90.9 %). Of those with advanced stage disease, all patients had cytoreductive surgery (100 %). Patient demographics and treatments received are detailed in Table 1.

**Table 1:** Demographics.

	All Cases (n = 166)	Quartile 1: Low Social Vulnerability (n = 35)	Quartile 2: Low-Medium Social Vulnerability (n = 39)	Quartile 3: Medium-High Social Vulnerability (n = 78)	Quartile 4: High Social Vulnerability (n = 14)
**Race: n (%)**					
White	152 (92.1)	34 (97.1)	37 (94.9)	68 (87.2)	14 (100.0)
Black	8 (4.8)	0 (0.0)	2 (5.1)	6 (7.7)	0 (0.0)
Other	6 (3.6)	1 (2.8)	0 (0.0)	4 (5.1)	0 (0.0)

**Insurance: n (%)**					
Medicare	64 (40.8)	14 (40.0)	20 (51.3)	25 (32.1)	5 (35.7)
Private	86 (54.8)	19 (54.3)	16 (41.0)	45 (57.7)	6 (42.9)
Medicaid	6 (3.8)	0 (0.0)	1 (2.6)	4 (5.1)	1 (7.1)
Other	10 (6.0)	2 (5.7)	2 (5.1)	4 (5.1)	2 (14.3)

**Stage: n (%)**					
Stage I	14 (8.6)	1 (2.9)	7 (17.9)	6 (7.7)	0 (0.0)
Stage II	14 (8.6)	3 (8.6)	2 (5.1)	8 (10.3)	1 (7.1)
Stage III	92 (56.4)	20 (57.1)	23 (58.9)	43 (55.1)	6 (42.9)
Stage IV	40 (24.5)	11 (31.4)	6 (15.4)	17 (21.8)	6 (42.9)
Unstaged/Unknown	6 (3.6)	0 (0.0)	1 (2.6)	4 (5.1)	1 (7.1)
**Age: median (range)**	61.7 (21.9–84.4)	63.4 (34.5–78.3)	62.3 (28.0–83.5)	59.8 (21.9–82.2)	60.0 (50.0–84.4)

**Genetic Testing Received**					
Yes	138 (83.1)	31 (88.6)	29 (74.4)	64 (82.1)	14 (100)
No	28 (16.9)	4 (11.4)	10 (25.6)	14 (17.9)	0 (0)
Unknown	0 (0)	0 (0)	0 (0)	0 (0)	0 (0)

**Chemotherapy Received**					
Yes	151 (90.9)	33 (94.3)	33 (84.6)	72 (92.3)	13 (92.9)
No	13 (7.8)	2 (0.06)	5 (12.8)	5 (6.4)	1 (7.1)
Unknown	2 (1.2)	0 (0)	1 (2.6)	1 (1.3)	0 (0)

**Optimal Cytoreductive Surgery Performed**					
Yes	156 (93.9)	33 (94.3)	36 (92.3)	74 (94.9)	13 (92.9)
No	10 (6.0)	2 (0.06)	3 (7.7)	4 (5.1)	1 (7.1)
Unknown	0 (0)	0 (0)	0 (0)	0 (0)	0 (0)

### 3.2 Time to treatment

There was a trend towards increased time from diagnosis to treatment amongst patients in the highest social vulnerability quartile (median 40, range 15 to 320 days) when compared to those in the low and low-medium social vulnerability quartiles (median 22 – 27, range 0 to 227 days) (Table 2). Patients in the highest quartile of social vulnerability had the longest time from diagnosis to treatment (median 40 days). In pairwise comparisons among social vulnerability quartiles, this was significantly different from patients in the medium–high social vulnerability quartile (median 26 days; p = 0.044), though when adjusted for multiple comparisons, this finding was no longer significant. Though not significant, there was also a time from diagnosis to treatment disparity between patients of the highest social vulnerability quartile and patients of the lowest social vulnerability quartile (median 27 days; p = 0.07) and low-medium social vulnerability quartile (median 22 days; p = 0.11).

When examining specific treatments received by these different groups of patients, patients in the low and medium–low social vulnerability quartiles were more likely to experience a shorter length of time to chemotherapy (median 29.5–32 days) when compared to patients in the medium–high and high social vulnerability quartiles (median 41 – 45 days). Patients in the highest social vulnerability quartile experienced the greatest length of time to surgery (median 128 days) amongst all quartiles. Patients in the low, medium–low, and medium–high quartiles experienced shorter times to surgery (median 82.5–98 days).

**Fig. 1:**
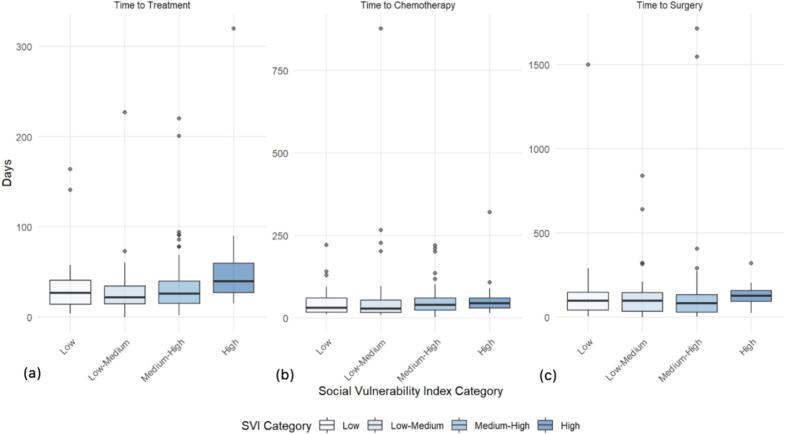
Boxplot time to treatment comparisons by social vulnerability index category. (a) Time to any treatment comparison (b) Time to chemotherapy comparison (c) Time to surgery comparison

Fig. 1: Social Vulnerability Index Quartile Comparisons of Time from Diagnosis to (a) Any Treatment, (b) Chemotherapy, and (c) Surgery.

Time to any treatment, time to chemotherapy, and time to surgery trended towards increasing as social vulnerability increased (Fig. 1). Amongst the four themes of social vulnerability, racial and ethnic minority status (p = 0.08) and household characteristics (p = 0.17) were the strongest contributors to this trend, as opposed to socioeconomic status (p = 0.36) and housing type and transportation (p = 0.34).

### 3.3 Time to palliative care consultation

**Fig. 2:**
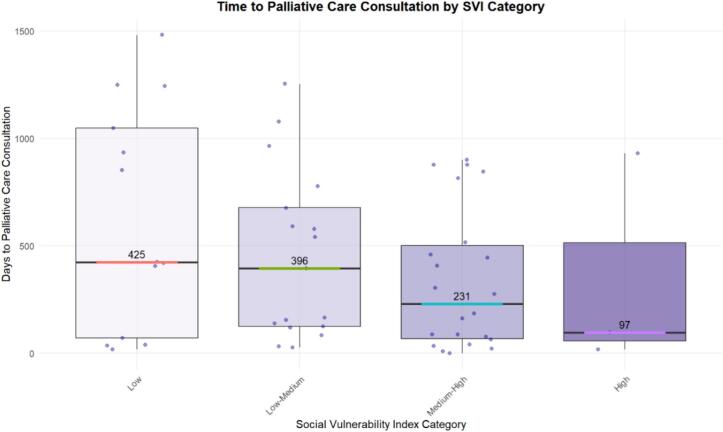
Boxplot time to palliative care comparisons by social vulnerability index category.

There was a trend towards shorter time from diagnosis to palliative care consultation for patients of higher social vulnerability. Patients in the low and medium–low social vulnerability quartiles experienced longer times to palliative care (median 396 – 425 days) as compared to patients in the high and medium–high social vulnerability quartiles (median 97 – 231 days) (Fig. 2).

Fig. 2: Social Vulnerability Index Quartile Comparisons of Time from Diagnosis to Palliative Care.

### 3.4 Progression-Free survival

There was no observed difference in progression-free survival between patients of different social vulnerability quartiles (p = 0.44).

**Fig. 3:**
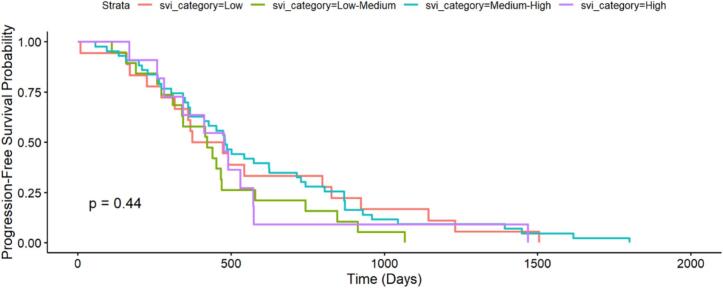
Cox proportional hazards model of progression-free survival according to social vulnerability index category.

Fig. 3: Progression-Free Survival by Social Vulnerability Index Category.

## 4 Discussion

In this retrospective cohort study, ovarian cancer patients with higher social vulnerability experienced longer times to treatment initiation with delays of up to 13 to 18 days. In subgroup analysis, primary contributors to this finding appear to be racial and ethnic minority status. Longer times to both surgery and chemotherapy were noted in patients with higher social vulnerability scores. In turn, shorter times to palliative care consultation were observed among patients with greater social vulnerability. There were no differences in progression-free survival according to social vulnerability. To our knowledge, this is the first study to assess the relationship between social vulnerability and access to treatment in ovarian cancer patients.

Patients with greater social vulnerability may have experienced longer times to treatment for various reasons, including delays with insurance coverage, difficulties with transportation, decreased health literacy, financial challenges, or unfavorable social circumstances. Social vulnerability captures the various, superimposed ways in which certain communities are systematically disadvantaged—this disadvantage appears to translate into clinically meaningful differences in time to treatment. Timely treatment is critical in the care of gynecologic malignancies.

Multiple studies have shown that social vulnerability predicts worse outcomes, as measured by progression-free survival and overall survival, in multiple cancers (Councell et al., 2024, Chen et al., 2023, Meier et al., 2024) and in ovarian cancer (Hicks et al., 2025, Borho et al., 2025). Other studies have revealed associations between delays to treatment and social vulnerability in other cancers (Zyczynski et al., 2022). Incorporating our study’s findings, it seems that delays to treatment might contribute to these differences in oncologic outcomes among ovarian cancer patients of differing social vulnerability status. Other studies have looked at deviations from guideline-concordant therapy as a measure of access to treatment and have found that black and lower socioeconomic status patients are less likely to receive adherent treatment (Akinyemiju et al., 2018). There are evidently several ways of defining access to care. In examining treatment delays, our study contributes to understanding an important dimension of access to care.

Palliative treatment is another integral part of ovarian cancer care given the disease’s notoriously high morbidity and mortality (Key Statistics for Ovarian Cancer, 2025, Surveillance and Program, 2018, Beesley et al., 2022, Bankhead et al., 2005). Our study revealed that patients with greater social vulnerability experienced shorter times to palliative care consultation; this may indicate that patients with higher social vulnerability more quickly progressed to debilitating symptoms that required specialized attention. Alternatively, prior studies also noted that inpatient settings neutralize racial/ethnic disparities in access to palliative care (Sharma et al., 2015). Still, socioeconomic inequities in access to palliative care have been noted by multiple studies (Møller et al., 2023, Nelson et al., 2021). A comparison of time to palliative care according to social vulnerability in patients matched by symptom burden or prognosis may elucidate the underlying reasons for the differences noted in this study and others.

Limitations include a smaller sample size with limited racial/ethnic diversity, as our cohort included primarily white women. Additionally, the data on overall survival in our patient cohort was not mature and thus not included. This may lead to an incomplete understanding of our patients’ oncologic outcomes. Our study, however, was designed to explicitly examine access to care in ovarian cancer rather than outcomes—our findings can be evaluated in context of the existing robust literature on disparities in oncologic and surgical outcomes among ovarian cancer patients (Hicks et al., 2025, Borho et al., 2025). Furthermore, given that social vulnerability is a zip-code level measure rather than a person-level measure, it may not adequately capture the various individual factors that mediate one’s ability to access care.

Our study also has several strengths. Our tertiary cancer center is an important care destination for ovarian cancer patients throughout the Southeastern United States. According to studies, Tennessee has higher average social vulnerability compared to the nation (Ng et al., 2025). Tennesee’s social vulnerability is further similar to or slightly higher than the average for the regional South, which has higher average social vulnerability scores as compared to the nation. This trend reflects persistent structural barriers in the South. The states in this region of the country largely did not opt into Medicaid expansion. Several studies have shown that Medicaid expansion is associated with better health outcomes (Guth et al., 2020). Thus this study offers an opportunity to understand the treatment patterns of patients in a particularly vulnerable region of our country. The usage of social vulnerability as a cumulative measure of relative disadvantage is yet another strength of this study. Effective health equity research necessitates an intersectional lens, one provided by measures such as social vulnerability.

Our study reveals that ovarian cancer patients with higher social vulnerability experience greater delays to care, an important dimension of access to healthcare. Several studies have shown that delays in treatment constitute an independent risk factor for poor ovarian cancer prognosis (Zhao et al., 2024). The impact of treatment delays upon increased mortality risk in various other cancers, including ovarian cancer, has been established (Zhao et al., 2024). Ovarian cancer patients who are more socially vulnerable face increased treatment delays and thus, related prognostic risk. Studies have shown that assistance with care coordination, proactive social risk screening, and linkage with appropriate resources may mitigate delays in cancer care in patients with high social vulnerability (Ruiz-Pérez et al., 2019, Horrill et al., 2024).

Future directions for study include assessing the relationship between treatment delays and the setting of gynecologic oncology referral and further examining whether treatment delays occurred prior to gynecologic oncology referral, after, or both. Given the limited diversity of our cohort, validating our findings in a more racially and ethnically diverse patient population will allow for a more comprehensive assessment of treatment disparities in ovarian cancer. Future studies may also consider assessing social vulnerability in a continuous rather than categorical manner as an alternative way of examining the data and eliciting trends.

Gynecologic oncologists should assess a patient’s risk for treatment delay and ensure the timely delivery of care. Social vulnerability, quickly measured using a patient’s zip code, may serve as a valuable tool for holistically evaluating a patient’s risk of delay. Social vulnerability can further be used on a policy level to identify higher-risk populations for treatment delays and prioritize them for tailored interventions.

## 5 Conclusions

In conclusion, this retrospective study revealed that ovarian cancer patients with higher social vulnerability experience longer times to treatment initiation and shorter times to palliative care consultation. Further study in more racially and ethnically diverse patient populations is warranted to expand upon our results.

## CRediT authorship contribution statement

**Nikita Bastin:** Writing – review & editing, Writing – original draft, Methodology, Funding acquisition, Data curation, Conceptualization. **Marc Robinson:** Data curation. **Amir Javid:** Formal analysis. **Lauren S. Prescott:** Writing – review & editing, Data curation. **Alaina J. Brown:** Writing – review & editing, Writing – original draft, Supervision, Methodology, Data curation, Conceptualization.

## Declaration of competing interest

The authors declare that they have no known competing financial interests or personal relationships that could have appeared to influence the work reported in this paper.
